# The prevalence of low back pain in Iranian dentists: An epidemiological study

**DOI:** 10.12669/pjms.332.11519

**Published:** 2017

**Authors:** Mohammad Ali Mohseni-Bandpei, Nahid Rahmani, Faezeh Halimi, Muhammad Nazim Farooq

**Affiliations:** 1 Dr. Mohammad Ali Mohseni-Bandpei, PT, PhD. Pediatric Neurorehabilitation Research Center, University of Social Welfare and Rehabilitation Sciences, Tehran, Iran. Visiting Professor, University Institute of Physical Therapy, Faculty of Allied Health Sciences, University of Lahore, Pakistan; 2 Dr. Nahid Rahmani, PT, PhD. Assistant Professor, Pediatric Neurorehabilitation Research Center, University of Social Welfare and Rehabilitation Sciences, Evin, Tehran, Iran; 3 Dr. Faezeh Halimi, MD. Resident of Gynecology, School of Medicine, Mashhad University of Medical Sciences, Mashhad, Iran; 4 Dr. Muhammad Nazim Farooq, PhD Scholar. Islamabad College of Physiotherapy, Rawalpindi, Pakistan

**Keywords:** Dentists, Low back pain, Prevalence, Risk Factors

## Abstract

**Objective:**

Low back pain (LBP) is one of the most common and prevalent work-related conditions. The aim of this study was to estimate the prevalence and risk factors associated with LBP in dentists and to analyze the association between individual and occupational characteristics and LBP.

**Methods:**

Following ethical approval, 300 dentists from Tehran Iran have voluntarily participated. Different questionnaires were completed to collect personal, occupational characteristics and the prevalence and risk factors of LBP. Visual analogue scale and Oswestry disability questionnaires were used to determine pain intensity and level of functional disability.

**Results:**

The results indicated that point, last month, last six month, last year and lifetime prevalence of LBP were 24.6%, 24.9%, 27.7%, 28.1% and 31.4%, respectively. A significant correlation was found between the prevalence of LBP and preventive strategies, general health condition, having an assistant and job satisfaction. Body mass index, age and gender were not significantly correlated with the prevalence of Low back pain

**Conclusions:**

The prevalence of Low back pain in dentists appears to be high. Further studies focusing on the effectiveness of different preventive strategies are recommended.

## INTRODUCTION

Low back pain (LBP) is one of the most common and prevalent work-related musculoskeletal disorders in many countries.^1^ The life time prevalence of LBP is reported to be high, affecting nearly 80% of people at some time in their adult life, and the point prevalence is ranging from 30% to 50%.^2^ For example, in France more than half of the French population experienced LBP at least one day in the previous 12 months.^3^ In Iran, LBP is reported to be the most common health problem affecting all population with different prevalence rates, ranging from 17% for school children,^4^ 31.1% for teachers,^5^ 62% for nurses^6^ and 84% for pregnant women^7^ to 84.4% for surgeons.^8^

In a German study, average total back pain cost per patient was estimated to be 1322 per year.^9^ Different reported prevalence rates may be attributed to different methodologies, definition for LBP, definition for point prevalence, small sample size, etc.

As dentists use prolonged sitting and standing during their job, apply awkward posture and repetitive movements, many loads are exerted to the lumbar spine. It is believed that the higher muscular demand may lead to fatigue and consequently increase the risk of LBP in dentists.^10^,^11^

In a systematic review, it was reported that the prevalence of general musculoskeletal pain ranges between 64% and 93% and the most prevalent regions for pain in dentists have been shown to be the back (36.3-60.1%).^10^ However, despite technical advances, dentists worldwide and particularly in the middle east are still at higher risk of developing LBP.^12^,^13^

The aim of this study was to estimate the prevalence and risk factors associated with LBP in dentists of Tehran, Iran and to analyze the association between individual and occupational characteristics and LBP.

## METHODS

### Study design and data collection

This cross sectional study was received approval from the medical ethics committee at the University of Social Welfare and Rehabilitation Sciences, Ministry of Health and Medical Education, Iran.. According to the sample size estimation formula for epidemiological studies and the 95% confidence interval, previous reported prevalence of 53%, and 10% error of prevalence rate, 341 subjects were needed which was rounded to 350. Three hundred and fifty dentists from Tehran Iran were randomly recruited. Dentists were included if they were qualified to work as a dentist, had at least one year of work experience and also were willing to participate in the present study. The exclusion criteria were: any history of spinal deformities (e.g. scoliosis), malignancies, rheumatoid arthritis, spondylolysis and spondylolisthesis, any metabolic and respiratory disease, any fracture or tumor, trauma to the lower back and osteoporosis. A written information sheet consisting of information about the aim and the purpose of the study were sent to participants and then they were asked to sign the consent form if they were willing to participate.

Different questionnaires were used to collect the individual and occupational information of the participants. The validated versions of Visual analogue scale (VAS)^14^ and Oswestry disability questionnaire (ODI)^15^ were used to determine the pain intensity and the level of functional disability, respectively. The individual and occupational questionnaires consisted of the information about age, height, weight, body mass index, marital status, level of education, years of work experience and working hours per day. Other questions were about the work loads, repetitive movements and using vibration during work, awkward postures, any types of treatment provided each day (surgery, root canal, filling and tooth extraction), prolonged positions (sitting or standing), medical history. All participants were asked to determine if they received any types of treatment. They were categorized into four groups, those being at rest, received medications, physiotherapy and exercises as well as surgery groups.

### Data Analyses

Statistical analysis was performed using SPSS (version 20) software (SPSS, Cary, NC). Categorical and numerical variables were studied using cross-tabulation with 95% confidence intervals and X^2^ analysis was used to examine the relationship between two or more variables. The level of statistical significance was set at 0.05.

## RESULTS

Of those original sample (N=350), 300 dentists returned the questionnaires (response rate of 86%). Two hundred and fifteen (71.7%) of participants were males and 85 (28.3%) were females. The sample characteristics are shown in Table-I and the epidemiological data collected from dentists is shown in Table-II. The management of LBP for those dentists who received treatment is reported in Table-III. The results of frequencies, Odd’s ratio and CIs for predictive factors of life time prevalence of LBP using a logistic regression model are presented in Table-IV. Point, last month, six month, last year and life time prevalence of LBP were 24.6%, 24.9%, 27.7%, 28.1% and 31.4%, respectively.

**Table-I:** Characteristics of dentists.

*Variables*	*Mean (SD)*	*Range*
Age (years)	41.30(8.43)	28–70
Height (cm)	175.83(8.77)	156–196
Weight (kg)	73.56(12.68)	49–102
BMI (kg/m^2^)	27.85(3.67)	18.90–39.01
Years of practice (year)	12.83(7.46)	1–42
Working hours per day (hour)	7.25(2.43)	2–16

BMI:Body Mass Index, SD:Standard Deviation.

**Table-II:** Low back pain prevalence of dentists.

*Period of prevalence*	*Prevalence rate*
Point prevalence	24.6%
Last month prevalence	24.9%
6 month prevalence	27.7%
Annual prevalence	28.1%
Lifetime prevalence	31.4%

**Table-III:** Management of low back pain received by dentists.

*Procedure/Treatment*	*No. (Percent)*
Rest	11(11.7)
Medication	19(20.21)
Physiotherapy and exercise	59(62.3%)
Surgery	5(5.31)

**Table-IV:** Odds ratio and CIs for predictive factors of life time prevalence of low back pain in dentists.

*Variabless*	*Frequency (%) of total sample*	*Frequency (%) affected by LBP*	*Odds Ratio*	*95% Confidence intervals*	*P-value*
***Gender***					
Male	215(71.7)	59(27.44)	1.24	0.61-1.25	0.292
Female	85(28.3)	35(41.17)			
***Age(y)***					
<40	125(41.7)	31(24.8)	1.11	0.77-1.33	0.411
41-50	83(27.6)	37(44.57)			
51-60	57(19)	19(33.33)			
>60	35(11.7)	7(20)			
***BMI***					
< 20	17(6)	2(11.77)	0.97	0.79-2.28	0.071
20-25	97(32)	25(25.78)			
25-30	156(52)	59(37.83)			
>30	30(10)	7(23.34)			
***General health***					
Reported healthy	211(70.3)	46(21.80)	7.14	3.88-11.14	0.000
Reported unhealthy	89(29.7)	48(53.94)			
***Years of practice***					
<10	103(34.3)	28(27.18)	0.62	0.56-1.09	0.49
10-20	129(43)	40(31.1)			
>20	68(22.7)	26(38.24)			
***Exercise***					
Not exercising	204(68)	68(33.33)	0.91	0.41-1.22	0.091
Exercising	96(32)	26(27.09)			
***Preventive Strategies (PS)***					
Without PS	108(36)	43(39.82)	3.01	1.92-5.11	0.022
With PS	192(64)	51(26.56)			
***Assistant***					
Without assistant	183(61)	75(84.27)	1.88	0.99-3.33	0.031
With assistant	117(39)	19(16.23)			
***Job satisfaction***					
No	11(3.6)	2(18.18)	1.09	0.39-1.06	0.000
Low	63(21)	23(36.51)			
Moderate	191(63.7)	65(34.03)			
High	35(11.7)	4(11.42)			

CI: Confidence Interval, BMI: Body Mass Index, PS: Preventive Strategies, LBP: Low Back Pain.

The mean and standard deviation of pain intensity of those dentists who suffered from LBP on VAS were 38.88±19.56 mm. The mean and standard deviation of functional disability on ODI were 39.56%±20.09%.

The results in Table-III shows that the female dentists were more likely to report LBP than men but this was not statistically significant (p=0.29). The results also demonstrated that older dentists (>50 years old) were more affected than younger ones but this did not reach to a statistically significant level (p=0.41). Similarly, there was no statistically significant correlation between body mass index (BMI) and prevalence of LBP (r=0.23, p=0.13).

According to the results provided in the Table-IV, a significant correlation was found between prevalence of LBP and general health, using preventive strategies, having an assistant and job satisfaction. There was no significant correlation between prevalence of LBP and years of practice (p = 0.49) but dentists who had more than 20 years job experience seem to be more at risk of developing LBP. The results indicated that prevalence of LBP was not significantly correlated to exercise performed by dentists (p=0.09).

## DISCUSSION

The results indicated that in the present study the lifetime prevalence of LBP was relatively high in Tehran Iran dentists. A significant correlation was found between the prevalence of LBP and preventive strategies, general health condition, having an assistant and job satisfaction. These results were consistent to the results of the previous epidemiological studies.^16^,^17^ Many studies have showed that dentists reported more pain in their neck and shoulders.^18^ Lower back was the second site of musculoskeletal disorder.

According to the results of the present study age, gender and BMI were not significantly correlated with the prevalence of LBP. But dentists aged between 41-50 years and older reported more pain. Similarly, in a study conducted in South Africa older women (>40 years) experienced more LBP than younger ones (<40 years).^19^

The previous studies showed that gender was a risk factor for LBP.^20^ Female dentists reported more pain in lower back than males but this was not statistically significant.^20^,^21^ The results of the present study were consistent with other studies which investigated the relationship between gender characteristics of dentists and prevalence of musculoskeletal disorders. For example Leijon O, Mulder M et alconcluded that female were more likely to report LBP than men.^22^ In contrast, Aasa et al. demonstrated that males reported a higher prevalence of LBP than women.^23^ The differences among studies might be due to the different definition of LBP and its symptoms and also population participated.

According to the results of the current study, there was no significant correlation between years of practice and prevalence of LBP but dentists who had more than 20 years of work experience reported more LBP. The results of the present study were similar to the results of the study carried out by Gaowgzehet al.^16^

The current study also demonstrated that preventive strategies were negatively associated with the LBP prevalence. Dentists who had not used any preventive strategies such as appropriate set of relaxation and stretching exercises, using assistive devices, using assistant, ergonomic guidance, evaluation of dental equipment and changing postures reported more LBP. Similar to the results of the present study, many studies have found significant correlation between prevalence of LBP and using preventive strategies.^24^ Also, those dentists who worked without an assistant reported more pain in their lower back. Previous studies have confirmed the results of present study and they explained that working without an assistant was a risk factor for prevalence of LBP.

In the present study job satisfaction was significantly correlated to LBP. The study conducted by Hoogendoorn et al. (2002) concluded that job satisfaction was a risk factor for sickness absence due to LBP.^25^ Another study carried out by Mohseni Bandpei et al. demonstrated the association between job satisfaction and LBP prevalence in nurses.^6^ Therefore, based on the results of the current study and the previous studies, job dissatisfaction was related to an increased risk for the occurrence of LBP.

However, it seems that addressing possible risk factors in different groups of high risk people for LBP and monitoring the risk factors would be an appropriate and effective way to reduce the impact of the problem.

### Limitations and future studies

The nature of cross-sectional studies does not provide a good basis for establishing causality and therefore, no causation can be implied in this study. Another limitation is the questionnaire used. Participants may not necessarily answer with perfect accuracy. This may magnify or minimize the effects of certain variables, affecting the study’s results. Apart from the limitations inherent to the design of the current study, another limitation of the current study was investigating the prevalence of LBP in dentists with different workloads. It seems that dentists with different levels of workloads might have different working demands and conditions that, in turn, may be a source of bias for the results of this study. The current study was performed on dentists in Tehran province, in Iran. It was assumed that the dentists participating in this study were a representative sample of dentists in Tehran province in Iran. However, this study used a small sample of dentists in only one province, which may not be representative of Iranian dentists. Future study should clearly address this concern. Although this study investigated the association between the prevalence of LBP and some risk factors, further study with larger and more homogenous sample is needed to confirm these associations. Also, future research with cohort or randomized controlled clinical trial designs should focus on the evaluation of different preventive strategies with a greater emphasis on monitoring the risk factors as well as evaluating the effect of ergonomic factors to reduce the impact of such a major health concern in dentists.

## CONCLUSIONS

The prevalence of LBP was relatively high in Iranian dentists. Prolonged sitting and standing, using bad posture and repetitive movements, doing work without any assistant and without rest, not applying any kinds of preventive strategies and using heavy instruments during dental works were identified as factors exerted abnormal loads to the spine and increased risks of musculoskeletal disorders in dentists.

### Authors’ Contribution

**MAMB:** Concept, design, data collection, statistical analysis, writing and approval of manuscript.

**NR:** Concept, design, data collection, statistical analysis, interpretation of data and writing of manuscript.

**FH:** Design, data collection, interpretation of data and writing of manuscript.

**MNF:** Concept, design, writing and approval of manuscript.
